# Distant mothering, grandparenting, intergenerational coparenting relationship, and child adjustment: evidence from Chinese families with young left-behind children

**DOI:** 10.3389/fpsyg.2025.1660162

**Published:** 2025-12-17

**Authors:** Ruwen Liang, Karla Van Leeuwen

**Affiliations:** 1 Normal College, Jimei University, Xiamen, China; 2 Parenting and Special Education Research Unit, KU Leuven, Leuven, Belgium

**Keywords:** coparenting, grandparenting, left-behind children, mothering, parenting

## Abstract

**Introduction:**

In China, it is a common practice for rural-to-urban migrant parents, due to working conditions, to leave their children with grandparents in their rural hometowns, and to raise their child from a distance (e.g., via mobile phone). Little is known about the association between (co-)parenting processes and child adjustment in these families. This cross-sectional study examined, in households with left-behind children (LBC), how positive grandparenting and distant mothering are associated with the child’s adjustment in home and preschool settings. The quality of the mother-grandparent coparenting relationship was hypothesized to be a moderator in this association.

**Methods:**

Questionnaire data were collected from 185 triads, namely preschool teachers (reporting child adjustment), grandparents (reporting child adjustment, grandparenting, and coparenting relationship quality), and migrant mothers (reporting coparenting relationship quality and distant mothering) of LBC aged 3 to 6.

**Results:**

Multiple hierarchical regression analyses showed that more positive grandparenting was associated with more child prosocial behavior and fewer difficulties. A significant interaction effect indicated that low coparenting relationship quality can be a risk factor: when mothers reported low levels of distant proactive control and the quality of the coparenting relationship was low, children showed less prosocial behavior.

**Discussion:**

A comprehensive family intervention program involving parents and grandparents at the same time may be a better option given the interrelatedness of (behaviors of) members within different family subsystems.

## 1 Introduction

The recent burgeoning number of studies on coparenting, which is defined as shared childrearing responsibility by caregivers (Feinberg, 2003), is indicative of a growing conviction on its significance to family functioning and child development (Teubert and Pinquart, 2010; Eira Nunes et al., 2021; Hawkins et al., 2022). Co-parents are not necessarily parents of a child but can also be grandparents. In Asian communities, intergenerational coparenting is a common practice, prompted by social values, the work situation of parents, and three generations living together (McHale et al., 2014; Bai et al., 2023). In China, large economic gaps between rural and urban regions motivate rural residents with limited financial means to seek employment opportunities and higher incomes in large cities. The work-related migration of parents often forces the separation of parent and child. This is especially the case for families who cannot afford the prohibitive expenses of moving together and who cannot break through the constraints of rural household registration (*hukou*; Ye and Pan, 2011). Many children are left behind by both parents in their rural hometowns and live without parental physical companionship even from an early age, which has been found to increase the risk of negative consequences for their development and well-being (Fellmeth et al., 2018; Hu et al., 2020; Bai et al., 2022). In that case, the in-hometown grandparents who live with these young children usually take over the parenting task from the parents.

In examining the associations between parenting and child outcomes in the families of left-behind children, many previous studies investigated the parenting practices of the primary caregivers, either an in-hometown parent or a grandparent (Wen and Lin, 2012; Yang and Liu, 2020; Pan et al., 2021), but marginalized the contributions from migrant mothers or fathers who try to parent their children from a distance. Besides the role of a family breadwinner, migrant mothers are reconstructing their caregiver identity in the digital era by interacting with separated family members through information and communications technologies like mobile phones. Distant parenting, defined as electronic-device-mediated remote childrearing behaviors through audio, video, and text communications, has become increasingly prevalent in these families (Gan et al., 2020; Tang et al., 2022; Liang et al., 2023). Compared to migrant fathers, migrant mothers revealed more intense involvement in mobile phone parenting (To et al., 2018; Li, 2022; Liang et al., 2023). Therefore, it is important to consider both grandparenting and parenting, especially distant mothering, when examining the ecological context of left-behind children’s early development.

Family systems theory suggests that the family as a system consists of family members who are part of different subsystems related to grandparenting, distant mothering, and coparenting (Minuchin, 1985; Cox and Paley, 1997). These subsystems, including grandparent–child, mother–child, and mother-grandparent relationships, intricately operate and influence individual family members’ behaviors (Krishnakumar and Buehler, 2000; Feinberg, 2003). This theoretical model is the basis for trying to understand how the quality of the mother-grandparent coparenting relationship perceived by migrant mothers and grandparents interacts with the parenting behaviors of these caregivers and the left behind children’s socioemotional and behavioral outcomes.

### 1.1 Grandparent-child subsystem: grandparenting and child adjustment

Being separated from their parents can be a dramatic change in the lives of young children, requiring them to adjust psychosocially to the physical absence of their parents. This adjustment can go well but can also sometimes lead to behavioral difficulties in the form of externalizing problems (e.g., hyperactivity symptoms or aggressive behaviors) and/or internalizing problems (e.g., symptoms of depression, anxiety) or reduced socio-emotional skills (Gilliom and Shaw, 2004; Carta et al., 2009).

With regard to child adjustment, Prevatt (2003) conceptualized and evaluated a risk and resilience model based on previous seminal research (Baumrind, 1967; Darling and Steinberg, 1993), in which different parenting behaviors of caregivers (parents and/or grandparents) protect or jeopardize child adaptation. In the meta-analysis by Pinquart (2017a, 2017b), positive parenting, including parenting dimensions such as warmth, behavioral control, autonomy granting, and the authoritative parenting style (i.e., affectively responsive parenting with high degrees of democratic participation and child regulation, Wu et al., 2002) are negatively associated, albeit in a small-sized manner, with child difficulties, while the associations for negative parenting like the parenting dimensions harsh control, psychological control, and the authoritarian parenting style (i.e., hostile parenting with high levels of unjustified punitive control, Wu et al., 2002), are the reverse.

Grandparents are ubiquitously the primary caregivers in families where both parents migrate for the sake of their jobs. They are especially influential in the immediate and direct environment around the children, and grandparenting thus plays a crucial role in young left-behind children’s adjustment. Many existing research on left-behind children and intergenerational caregiving in China has largely emphasized risks (such as prolonged separation, inconsistent caregiving, and overindulgent parenting) and has often portrayed grandparent care primarily as a source of problems (Gu, 2022; Shan et al., 2025). Indeed, previous studies have shown that children who are raised entirely by grandparents because of their parents’ migration generally display more maladaptive and fewer prosocial behaviors compared to parent-raised non-left-behind children (Fan et al., 2010; Fellmeth et al., 2018; Hu et al., 2020), this does not mean grandparent childrearing per se undermines child development. Some researchers argued that these differences are possibly related to poverty and parental absence instead of whether the caregiver is a grandparent (Li et al., 2019; Yang and Liu, 2020). A limited number of studies have examined how grandparenting dimensions or styles are related to child adjustment outcomes (Weissman et al., 2016; Hou et al., 2019; Li et al., 2019; Yang and Liu, 2020). As summarized above, prior work has suggested that both supportive and suboptimal grandparenting practices may be linked to children’s emotional and behavioral problems, but the findings regarding grandparental warmth are mixed. For instance, Li et al. (2019) found that suboptimal parenting practices by both grandparents and parents increased the risk of emotional and behavioral problems in Chinese adolescents, and reported a positive association between grandparental warmth and child difficulties, whereas other studies have documented negative associations between grandparental warmth and child difficulties (e.g., Hou et al., 2019; Yang and Liu, 2020). Thus, existing evidence on the role of grandparental warmth in child adjustment is inconsistent. While this risk-focused work is important, the risk–resilience literature also highlights the need to identify protective processes that help children adapt under adversity. In the present study, we therefore adopt a strengths-based perspective and focus on positive/authoritative grandparenting (alongside distant maternal responsivity) as a potential protective caregiving resource in high-risk left-behind families, rather than attempting to model the full range of grandparenting styles. Based on the literature, we formulated the following hypothesis:

*Hypothesis 1.* Higher levels of positive grandparenting are associated with fewer child difficulties and more prosocial behavior.

### 1.2 Mother–child subsystem: distant mothering and child adjustment

In face-to-face contexts, when only parenting by mothers is examined (and parenting by fathers is not), the associations generally remain the same (negative associations between positive parenting and child difficulties, and positive associations between negative parenting and child difficulties) as the meta-analysis findings by Pinquart (2017a, 2017b). Moreover, more positive and less negative mothering has been found to be associated with stronger prosocial and emotional competence of the child (Garner, 2006; Wang et al., 2006; Pastorelli et al., 2016).

As with general parenting, it is assumed that the special context of distant mothering also has an impact on child functioning (Madianou and Miller, 2011; Nichols and Selim, 2022; Tang et al., 2022). To be able to operationalize distant mothering, new measures for distant parenting have been developed (Weisskirch, 2009; Jensen et al., 2021; Liang and Van Leeuwen, 2024). Within our larger research project, an initial qualitative inquiry with migrant parents (Liang et al., 2023) identified six recurrent dimensions of mobile phone parenting. A subsequent quantitative study (Liang and Van Leeuwen, 2024) validated this six-factor structure (i.e., responsivity, autonomy granting, psychological control, proactive control, directive control, and harsh punitive control) in a sample of LBC’s parents. In that study, responsivity, autonomy granting, and proactive and directive control were positively intercorrelated, whereas psychological control and harsh punitive control clustered together and were negatively related to the more positive dimensions. This pattern mirrors distinctions in the general parenting literature between more supportive, structured practices and more intrusive or harsh forms of control.

From this perspective, distant responsivity, autonomy granting, and proactive and directive control can be viewed as reflecting relatively positive and structured distant mothering, whereas psychological and harsh punitive control reflect more negative distant mothering. However, despite the initial conceptualization and psychometric validation of these six dimensions, little is known about how they are empirically related to child prosocial behavior and difficulties, or whether distant mothering makes a unique contribution to left-behind children’s socio-emotional outcomes beyond grandparenting. Therefore, in the present study distant mothering is our primary focal construct, whereas grandparenting is included as a broader indicator of positive caregiving in the home context rather than being decomposed into multiple dimensions. Based on the literature on general mothering and child adjustment and on our prior work on distant parenting, we formulated the following hypothesis:

*Hypothesis 2.* Maternal parenting dimensions explain extra variance in child difficulties and prosocial competence after considering the contribution of positive grandparenting. We expect more maternal responsiveness, autonomy granting, proactive and directive control, and less psychological and harsh punitive control to be associated with fewer child difficulties and more prosocial behavior.

### 1.3 Mother-grandparent subsystem: coparenting relationship quality

Coparenting relationship quality refers to the extent to which parenting partners coordinate their childrearing efforts and support or undermine one another in parenthood (McHale, 1997; Feinberg, 2003). Boričević Maršanić and Kušmić (2013) concluded in their review that coparenting is a multidimensional concept including discrepancies in parental involvement and supportive/hostile-competitive aspects. In the comprehensive model proposed by Feinberg et al. (2012), coparenting relationship quality is defined in terms of coparenting agreement, coparenting closeness, exposure of child to conflict, coparenting support, coparenting undermining, endorsement of partner’s parenting, and division of labor. While most studies examine parental coparenting relationships within Chinese nuclear families (McHale et al., 2000; Ren and Xu, 2019; Fan and Chen, 2020; Liu et al., 2021; Wang et al., 2022) there is an increase in research into the mother-grandparent coparenting relationship (Li and Liu, 2019; Li et al., 2020; Luo, 2022; Bai et al., 2023; Xu et al., 2023).

The specific associations between coparenting relationship quality and child adjustment have been demonstrated in two meta-analytic studies, in which fewer children’s problem behaviors and better social functioning were significantly associated with a favorable coparenting relationship, but the effect sizes were small (Teubert and Pinquart, 2010; Zhao et al., 2022). The associations have been especially confirmed in Chinese families where fathers and mothers jointly parent their preschool-age children (Lam et al., 2018; Ren and Xu, 2019). Also, more recent studies on children in China have explored the associations between intergenerational coparenting relationship quality and children’s socio-emotional outcomes. For example, the study by Li and Liu (2019) on Chinese young children suggested that mother-grandparent coparenting relationship quality positively predicted the social competence of preschoolers. Another study further found that the effects of intergenerational coparenting relationship quality on social competence of preschoolers differed in strength between mother-maternal grandmother and mother-paternal grandmother coparenting patterns, with maternal grandmothers’ perception of coparenting relationship quality as a stronger predictor (He et al., 2023). While these studies have provided emerging evidence in urban co-residing family members, it is still unknown whether the dynamics of distant coparenting are similarly linked to the social–emotional outcomes of rural left-behind children. This leads us to the following hypothesis in the current study:

*Hypothesis 3.* Higher quality of the mother-grandparent coparenting relationship is related to fewer child difficulties and higher prosocial competence after controlling for positive grandparenting and distant mothering indicators.

### 1.4 Interrelated family subsystems: the moderating role of coparenting relationship quality in the associations between (grand)parenting and child adjustment

In the ecological model of coparenting, Feinberg (2003) posits a high-quality coparenting relationship as a moderator to buffer or protect children from the influence of adverse contexts (e.g., negative parenting). It is also reasonable to assume that a good coparenting relationship can facilitate the benefits children derive from nurturing environments (e.g., positive parenting). Indeed, initial findings have shown the moderating role of the coparenting relationship quality in the association between (grand)parenting and child outcomes (Scrimgeour et al., 2013; Latham et al., 2017; Yan et al., 2021; Zhang et al., 2022). For instance, as regards the facilitative model, Scrimgeour et al. (2013) found that in 58 two-parent American families, the interaction of maternal inductive reasoning (assessed when the child was 24 months) and cooperative coparenting (assessed when the child was 42 months) positively predicted the prosocial behavior of four-year-old children. To our knowledge, the moderating role of mother-grandparent coparenting relationship in the link between (grand)parenting and early childhood adjustment has rarely been investigated. Nevertheless, we can draw inspiration for a hypothesis in the current study, from a cross-sectional study on parent-grandparent coparenting in a sample of Chinese adolescents. Zhang et al. (2022), who included both coparenting relationship and parent-grandparent relationship as moderators, reported that the positive association between low-supportive grandparenting and adolescent depressive symptoms was significant when the adolescents perceived high quality of the parent-grandparent relationship, but at the same time less coparental cooperation. We thus hypothesize:

*Hypothesis 4.* Coparenting relationship quality moderates associations between (grand)parenting and child adjustment. To be specific, the links between higher levels of positive grandparenting and fewer child difficulties or more prosocial competence are expected to be stronger in children from families that perceive a better quality of mother-grandparent coparenting relationship. More maternal responsivity, autonomy granting, proactive control, directive control, and less psychological and harsh punitive control are more strongly associated with fewer child internalizing/externalizing problems and a higher prosocial competence when the children’s families report higher quality of intergenerational coparenting relationships.

### 1.5 The current study

The present cross-sectional study examined how three interrelated family subsystems (positive grandparenting, mobile-phone-based distant mothering, and the quality of the mother-grandparent coparenting relationship) are associated with child adjustment (i.e., prosocial behavior and difficulties) among Chinese families with young left-behind preschoolers in rural areas. Extending prior research on Chinese intergenerational parenting, which has mainly focused on grandparent caregiving in co-residing three-generation households (See Bai et al., 2023, for a review), we focus on families in which both parents have migrated, grandparents are the primary daily caregivers, and mothers engage in parenting from a distance via mobile phones. We conceptualize maternal parenting at a distance as a full parenting context, assessing multiple dimensions of distant mothering (responsivity, autonomy granting, proactive and directive control, psychological control, and harsh punitive control) alongside positive grandparenting.

This study may contribute in two main ways. First, it advances theoretical work by synthesizing and extending existing models. We integrate the risk/resiliency model of child adjustment (Prevatt, 2003) and the ecological model of coparenting (Feinberg, 2003) within a family systems framework (Minuchin, 1985; Cox and Paley, 1997), and extend these models to a translocal context in which caregivers share childrearing responsibilities despite long-term physical separation and intensive media use. In particular, we investigate whether the quality of the mother-grandparent coparenting relationship moderates associations between (grand)parenting behaviors and child outcomes in these digitally connected but geographically separated families. In doing so, we address a key gap in previous LBC-related studies, which have largely focused on grandparenting alone and have overlooked the contribution of distant parenting and its interaction with grandparent caregiving (Hou et al., 2019; Yang and Liu, 2020; Pan et al., 2021).

Second, this study enriches the empirical literature on intergenerational parenting in China by focusing on young LBC in an economically less developed rural region and by employing a multi-informant triadic design. In contrast to most previous intergenerational coparenting studies that relied on urban families and single- or two-informant designs (Hou et al., 2019; Li et al., 2019; Danielsbacka et al., 2022; Xu et al., 2022; Zhang et al., 2022), we combine reports from preschool teachers (child adjustment), co-resident grandparents (grandparenting and coparenting), and migrant mothers (distant mothering and coparenting). The findings can inform family-focused interventions by highlighting the need to consider positive grandparenting, distant mothering practices, and intergenerational coparenting quality together when promoting the well-being of LBC.

## 2 Methods

### 2.1 Participants and procedures

Triadic data were obtained from a cross-sectional online/telephone survey administered to teachers from 32 rural preschools, migrant mothers, and grandparents of 185 preschool-age left-behind children in Chuxiong Yi Autonomous Prefecture, Yunnan, a province with lower levels of economic development located in the southwestern region of China. According to official statistics (Chuxiong Prefecture Bureau of Statistics, 2023), approximately 37.0% of the local population belong to ethnic minority groups, predominantly Yi (29.7%); however, we did not record individual participants’ ethnic identity, and the ethnic composition of our sample may therefore differ from the population distribution. The present quantitative study is a component of a larger mixed-methods project, for which an initial exploratory qualitative study on mobile phone parenting and intergenerational coparenting in LBC’s families (Liang et al., 2023) provided in-depth insights that methodologically and theoretically informed the focus of the current quantitative investigation. Inclusion criteria were: families (a) where both parents were physically absent; (b) that were experiencing a three-month-above parent–child separation with at least one child aged three to six, left in the hometown; (c) where the grandparents served as primary caregivers; (d) where the mothers had regular communication with their children through mobile phones; and (e) where both grandparents and mothers were willing and able to participate in questionnaires or interviews. Most of the participating grandparents who primarily co-parented the children with migrant mothers, were female. The sample included 55.14% paternal grandmothers, 25.41% maternal grandmothers, 9.19% paternal grandfathers, and 10.27% maternal grandfathers. Migrant mothers were on average in their thirties (*M*_age_ = 31.94, *SD* = 3.76) and 91.35% of them engaged in occupations such as non-technical, semi-technical, or technical work. About 33.51% of the children were being separated from their mothers for 2 years or more. Table 1 presents extra demographic characteristics of the families involved in this study.

**Table 1:** Demographic information of the families and descriptive statistics.

Variables	*N*	Percentage
Child sex		
Girl	83	44.86
Boy	102	55.14
Family annual income (CNY)		
35,000 and below	10	5.41
35,001–55,000	21	11.35
55,001–75,000	43	23.24
75,001–95,000	63	34.05
95,001–115,000	31	16.76
115,001 and more	17	9.19
Father-child telecommunication frequency		
Less than once a week	54	29.19
Once a week	40	21.62
Twice a week	39	21.08
Three times a week	21	11.35
Four times a week	15	8.11
Five times a week	14	7.57
Six times a week and more	2	1.08

	Mean	SD
Child age	4.23	1.01
Mother’s years of completed education	9.75	1.87
Teacher-reported prosocial behavior	6.92	2.06
Teacher-reported child difficulties	12.35	4.76
Grandparent-reported prosocial behavior	7.10	2.06
Grandparent-reported child difficulties	10.41	4.59
Composed prosocial behavior	7.01	1.69
Composed child difficulties	11.38	4.06
Maternal responsivity	3.85	0.74
Maternal proactive control	3.70	0.95
Maternal directive control	3.37	0.90
Maternal autonomy granting	3.65	0.92
Maternal psychological control	2.31	0.93
Maternal harsh punitive control	1.63	0.53
Mother-reported coparenting relationship quality	4.52	0.67
Grandparent-reported coparenting relationship quality	4.77	0.56
Composed coparenting relationship quality	4.65	0.54
Positive grandparenting	3.73	0.71

SD, standard deviation; 1 CNY ≈ 0.14 EUR.

The data collection took place from May 2022 to January 2023. In light of COVID-19 restrictions, all questionnaires were completed remotely to ensure the safety of participants and to adhere to local public health guidelines. During this period, the involved preschools and participants were not severely affected by the coronavirus pandemic and still operated as usual except for stricter control on outsider access and heightened precautions. Mothers and classroom teachers received the links to the electronic questionnaires by messages. Teachers reported the target children’s behaviors on the Strengths and Difficulties Questionnaire online. Mothers completed a demographic questionnaire, the Mobile Phone Parenting Practice Questionnaire, and the Coparenting Relationship Scale. Investigators conducted telephone interviews to collect the answers from a grandparent who was the primary caregiver in each target family on the Strengths and Difficulties Questionnaire, Parenting Styles and Dimensions Questionnaire, and Coparenting Relationship Scale. All participants were compensated for their involvement by remuneration. In the beginning, we received a total of 216 teacher questionnaires. Out of the 18 teacher questionnaires that involved two children from the same families, only one of the children was selected. Among the participants, 22 triads had missingness of either the mother questionnaires or grandparent questionnaires. Consequently, we kept the 185 triads with complete information.

The study was approved and carried out following the ethical requirements of the Social and Societal Ethics Committee of KU Leuven (Approval number: G-2021-4012-R2(MAR)). Informed consent was obtained from all the mothers, grandparents, and teachers.

### 2.2 Measures

#### 2.2.1 Child adjustment

The Strengths and Difficulties Questionnaire (SDQ, Chinese version) measured children’s strengths and social–emotional-behavioral problems (Goodman, 1997). The 25-item SDQ consists of five five-item subscales including emotional symptoms (e.g., “Often unhappy, down-hearted or tearful”); conduct problems (e.g., “Steals from home, school or elsewhere”); hyperactivity/inattention (e.g., “Restless, overactive, cannot stay still for long”); peer relationship problems (e.g., “Picked on or bullied by other children”); and prosocial behavior (e.g., “Considerate of other people’s feelings”). We adopted an alternative two-subscale division that indicated not high but acceptable reliability in the studies using the SDQ (Du et al., 2008): child difficulties (an average score of the subscales with emotional, peer relationship, conduct, and hyperactivity problems, 20 items, *α* = 0.72 for grandparents’ report and *α* = 0.68 for teachers’ report), and prosocial behavior as indicator for a child’s strengths (*α* = 0.72 for grandparents’ report and *α* = 0.62 for teachers’ report). Participants responded to each item using a three-point rating format (0 = *not true*, 1 = *somewhat true*, 2 = *certainly true*). Higher scores indicated either higher levels of child maladaptation (difficulties) or positive adaptation (prosocial behavior). The correlations for child difficulties and prosocial behavior between reports by teachers and grandparents were 0.51 (*p* < 0.001) and 0.35 (*p* < 0.001) respectively. To reduce the number of analyses and avoid the type I error, we used an average (composite) score based on teachers’ and grandparents’ ratings.

#### 2.2.2 Distant mothering

The Mobile Phone Parenting Practices Questionnaire (MPPPQ; Liang et al., 2023) measures distant parenting dimensions among migrant parents of young children. Mothers were asked to report their parenting through mobile phones using a five-point response form (1 = *never* to 5 = *always*). This 47-item instrument includes six subscales, with (a) the responsivity subscale capturing mother’s recognition of children’s needs and their providing of nurturing and supportive responses (19 items, e.g., “I remotely notice and identify the current feelings of my child”); (b) the autonomy granting subscale indicating how mothers respect and encourage the independence of their children (five items, e.g., “I respect my child’s decision of calling me or answering the call”); (c) the proactive control subscale measuring mothers’ reasonable, appropriate, and proactive regulation (eight items, e.g., “I clearly explain my educational or behavioral expectations toward my child”); (d) the directive control subscale reflecting mothers’ demandingness for child obedience (five items, e.g., “I ask my child to be subordinate to his/her teacher”); (e) the psychological control subscale assessing the degree to which mothers intrude children’s thoughts, self-expression, and feelings (six items, e.g., “I tell my child that his/her performance is a waste of my hard work for him/her”); (f) the harsh punitive control subscale evaluating mothers’ remote physical coercion or mother-required corporal punishment conducted by a caregiver (four items, e.g., “I remotely ask my child to punitively stand”). In the current study, the Cronbach’s alphas were 0.95 for responsivity, 0.89 for autonomy granting, 0.85 for psychological control, 0.93 for proactive control, 0.81 for directive control, and 0.85 for harsh punitive control. We computed the mean score of each subscale where a greater score refers to more frequent parenting behaviors within this dimension.

#### 2.2.3 Positive grandparenting practices

We used the 26-items Parenting Styles and Dimensions Questionnaire (PSDQ) developed by Robinson et al. (2001) and validated by Wu et al. (2002) among Chinese respondents. The PSDQ consists of six subscales involving warmth/acceptance (seven items, e.g., “Gives praise when child is good”); reasoning/induction (four items, e.g., “Encourages child to talk about consequences of behaviors”); democratic participation (four items, e.g., “Takes child’s desire into account before asking to do something”); verbal hostility (three items, e.g., “Yells or shouts when child misbehaves”), physical coercion (five items, e.g., “Slaps when child misbehaves”), and nonreasoning/punitive (three items, e.g., “Punishes by taking privileges away with little explanation”). All items used a five-point response format (1 = *never* to 5 = *always*). To capture positive grandparenting, we adopted the authoritative parenting subscale (i.e., the combination of warmth, democratic participation, and reasoning subscales), with a higher mean score suggesting more positive/authoritative grandparenting. Given our focus on protective caregiving and the limited sample size, we used this composite as a single summary indicator of positive grandparenting rather than including all PSDQ positive and negative subscales as separate predictors in the models. The Cronbach’s alpha of the authoritative parenting subscale was 0.89.

#### 2.2.4 Quality of mother-grandparent coparenting relationship

We used a brief 14-item Chinese version of the Coparenting Relationship Scale (CRS), adapted by Luo (2022) and her colleagues from the original scale by Feinberg et al. (2012), to measure the quality of the mother-grandparent coparenting relationship. Two items from each of the following subscales (coparenting agreement, coparenting closeness, exposure of child to conflict, coparenting support, coparenting undermining, endorsement of partner’s parenting, and division of labor between mothers and grandparents) are included in this short version (e.g., “The child’s grandparent and I have the same goals for the child” and “I feel close to the child’s mother when I see her play with the child.”). All items for mothers and grandparents were consistent but worded differently (e.g., “The child’s mother and I have the same goals for the child” for the grandparent version and “The child’s grandparent and I have the same goals for the child” for the mother version) with a seven-point response scale (0 = *not true of us/never*, 6 = *very true of us/very often*). Cronbach’s alphas were 0.85 for mothers and 0.86 for grandparents. After adjusting the reversed-phrasing items, the mean scores of the mother-report and grandparent-report questionnaires were summed and averaged. The correlation between mother-perceived and grandparent-perceived coparenting relationship quality was 0.56 (*p* < 0.001), and to limit the number of variables, we created a composite coparenting score by calculating the mean of mothers’ and grandparents’ CRS ratings. Higher scores indicate good quality of coparenting relationships.

#### 2.2.5 Covariates

Child age and sex, family income, as well as maternal education background, were included as covariates since they are risk/protective factors related to child adjustment (Pinquart, 2017a, 2017b; Mensch et al., 2019). Although distant fathering was less documented in previous investigations, L. Wang et al. (2021) argued that paternal involvement, albeit rather low, was still found to matter for early childhood development in rural China. Therefore, this current study additionally adopted the frequency of contact with fathers per week as a covariate, which indicated the intensity of distant fathering.

### 2.3 Data analysis

We tested hypotheses *H1*, *H2*, and *H3* with hierarchical multiple regression analyses. We sequentially entered positive grandparenting, six distant mothering dimensions, and intergenerational coparenting relationship quality into the models with the covariates to examine to what extent these added variables explained variance in child adjustment. The same models were tested twice, once for child difficulties and once for prosocial behavior as dependent variables.

Using the “processR” package in R (Hayes, 2017), we also examined two-way interaction effects (i.e., positive quality of grandparenting × coparenting, and maternal parenting dimensions × quality of coparenting relationship) to test hypothesis *H4*. If a significant moderator was detected, a subsequent *post hoc* probing method was carried out to examine and interpret the interaction (Aiken et al., 1991). The first step was conducting separate regression analyses for three levels of the moderator (i.e., 1 *SD* above the mean, mean, 1 *SD* below the mean) and plotting the three simple regression lines. Next, conditional effects of the independent variables at the high, mean, and low values of the moderators were examined with *t* tests.

## 3 Results

### 3.1 Descriptive statistics and correlations regarding all variables

Table 1 provides the descriptive statistics of parents’ and grandparents’ characteristics, covariates, and child outcomes. Spearman correlation was used to examine the associations between child sex (0 = girl, 1 = boy) and other variables while the other bivariate associations among the key variables were tested with Pearson correlations. In Table 2, the correlations among all variables showed that child prosocial behavior was positively correlated to family income, frequencies of father-child telecommunication, positive grandparenting, the quality of mother-grandparent coparenting relationship, maternal responsivity, and maternal autonomy granting with small to medium effect sizes based on Cohen’s criteria (Cohen, 1988). In contrast, child difficulties had a negative, weak-to-medium association with mother’s completed educational years, family income, frequencies of father-child telecommunication, positive grandparenting, coparenting relationship quality, maternal responsivity, maternal autonomy granting, maternal proactive and directive control, and had a positive, weak-to-moderate association with maternal psychological control and harsh punitive control, respectively.

**Table 2:** Intercorrelations among all variables.

Variables	1	2	3	4	5	6	7	8	9	10	11	12	13	14
1. Child age														
2. Child sex	−0.15^*^													
3. Mother’s completed educational years	0.03	−0.05												
4. Family income	0.10	−0.05	0.22^**^											
5. Father-child telecommunication frequency	−0.06	−0.09	0.01	0.07										
6. Child prosocial behavior	0.03	−0.08	0.14	0.19^**^	0.16^*^									
7. Child difficulties	0.02	−0.09	−0.17^*^	−0.17^*^	−0.16^*^	−0.29^***^								
8. Coparenting relationship quality	0.01	−0.02	0.11	0.07	0.10	0.23^**^	−0.22^**^							
9. Positive grandparenting	−0.08	−0.05	0.07	−0.03	0.07	0.25^***^	−0.28^***^	0.15^*^						
10. Maternal responsivity	0.16^*^	−0.04	0.22^**^	0.13	0.04	0.23^**^	−0.24^***^	0.18^*^	0.10					
11. Maternal proactive control	0.03	0.00	0.15^*^	0.05	0.00	0.11	−0.21^**^	0.04	0.02	0.36^***^				
12. Maternal directive control	0.09	−0.04	0.13	0.13	0.00	0.06	−0.15^*^	0.09	−0.04	0.26^***^	0.35^***^			
13. Maternal autonomy granting	0.02	0.08	0.23^**^	0.20^**^	0.01	0.16^*^	−0.24^***^	0.12	0.16^*^	0.50^***^	0.35^***^	0.35^***^		
14. Maternal psychological control	−0.22^**^	−0.04	−0.11	−0.10	−0.08	−0.09	0.17^*^	−0.05	−0.10	−0.44^***^	−0.36^***^	−0.24^**^	−0.35^***^	
15. Maternal harsh punitive control	−0.06	−0.02	−0.18^*^	−0.27^***^	−0.03	−0.07	0.21^**^	−0.07	−0.01	−0.49^***^	−0.38^***^	−0.28^***^	−0.46^***^	0.41^***^

^*^p < 0.05, ^**^p < 0.01, ^***^p < 0.001. Spearman correlation was used for associations involving child sex while the other bivariate associations among the variables were tested by Pearson correlation. Child sex: 0 = girl, 1 = boy. The variables “child prosocial behavior” and “child difficulties” were composed based on teachers’ and grandparents’ ratings. The variable “coparenting relationship quality” was the quality of mother-grandparent coparenting relationship, composed by mothers’ ratings and grandparents’ ratings.

### 3.2 Hierarchical multiple linear regression analyses

Table 3 presents the results of hierarchical multiple linear regression analyses with positive grandparenting, dimensions of distant mothering, and the quality of mother-grandparent coparenting relationship as the independent variables and child prosocial behavior and difficulties as the dependent variables.

**Table 3:** Hierarchical multiple linear regression analyses regarding positive grandparenting, distant mothering, and coparenting relationship quality as the independent variables and child prosocial behavior and difficulties as the dependent variables.

Variables	Child prosocial behavior									Child difficulties								
	Model_1a			Model_2a			Model_3a			Model_1b			Model_2b			Model_3b		
	*β*	*SE*	*p*	*β*	*SE*	*p*	*β*	*SE*	*p*	*β*	*SE*	*p*	*β*	*SE*	*p*	*β*	*SE*	*p*
Child age	0.04	0.07	0.611	0.02	0.07	0.820	0.02	0.07	0.816	−0.01	0.07	0.851	0.01	0.07	0.902	0.01	0.07	0.903
Child sex	−0.03	0.07	0.637	−0.03	0.07	0.706	−0.03	0.07	0.687	−0.13	0.07	0.066	−0.13	0.07	0.073	−0.12	0.07	0.074
Mother’s years of completed education	0.08	0.07	0.277	0.04	0.07	0.544	0.04	0.07	0.617	−0.13	0.07	0.075	−0.08	0.07	0.287	−0.07	0.07	0.331
Family income	0.17	0.07	0.020^*^	0.18	0.07	0.017^*^	0.17	0.07	0.020^*^	−0.15	0.07	0.036^*^	−0.12	0.07	0.098	−0.12	0.07	0.111
Father-child telecommunication frequency	0.13	0.07	0.073	0.13	0.07	0.078	0.11	0.07	0.108	−0.15	0.07	0.038^*^	−0.14	0.07	0.042^*^	−0.13	0.07	0.058
Positive grandparenting	0.24	0.07	<0.001^***^	0.23	0.07	0.002^**^	0.21	0.07	0.005^**^	−0.28	0.07	<0.000^***^	−0.27	0.07	<0.000^***^	−0.25	0.07	<0.001^***^
Maternal responsivity				0.21	0.09	0.024^*^	0.18	0.09	0.046^*^				−0.10	0.09	0.233	−0.08	0.09	0.344
Maternal proactive control				0.01	0.09	0.873	0.01	0.09	0.891				−0.02	0.09	0.821	−0.02	0.09	0.837
Maternal directive control				0.07	0.08	0.396	0.07	0.08	0.357				−0.11	0.08	0.165	−0.12	0.08	0.145
Maternal autonomy granting				−0.01	0.08	0.943	−0.02	0.08	0.843				−0.06	0.08	0.439	−0.05	0.08	0.507
Maternal psychological control				0.04	0.08	0.632	0.03	0.08	0.694				−0.02	0.08	0.846	−0.01	0.08	0.909
Maternal harsh punitive control				0.11	0.09	0.213	0.11	0.09	0.225				0.04	0.09	0.612	0.05	0.09	0.582
Coparenting relationship quality							0.14	0.07	0.045^*^							−0.13	0.07	0.070
*df*	178			172			171			178			172			171		
*R*^2^	0.13			0.17			0.18			0.16			0.21			0.23		

^*^p < 0.05, ^**^p < 0.01, ^***^p < 0.001. SE, standard error. Child sex: 0 = girl, 1 = boy.

#### 3.2.1 Positive grandparenting as an independent variable

The models including positive grandparenting only, along with the covariates, explained 13% of the variance of prosocial behavior (*F_Model_1a_* (6, 178) = 4.36, *p* < 0.001) and 16% of the variance of child difficulties (*F_Model_1b_* (6, 178) = 5.69, *p* < 0.001). Positive grandparenting was consistently positively associated with child prosocial behavior (*β* = 0.24, *p* < 0.001; *β* = 0.23, *p* = 0.002; *β* = 0.21, *p* = 0.005; respectively in the three models) and also consistently negatively associated with child difficulties (*β* = −0.28, *p* < 0.001; *β* = −0.27, *p* < 0.001; *β* = −0.25, *p* < 0.001; respectively in the three models) even after adding other variables of mothering and coparenting.

#### 3.2.2 Dimensions of distant mothering as independent variables

First, among the six dimensions of distant mothering, prosocial behavior was significantly associated with maternal responsivity only (*β* = 0.21, *p* = 0.024). This association was still present after including the quality of coparenting relationship (*β* = 0.18, *p* = 0.046), suggesting that children acted more prosocially when migrant mothers were more responsive in their remote communications. However, the contribution of overall distant mothering variables to the variance of child prosocial behavior was not significant (Δ*R^2^* = 0.04, *p* = 0.262), over and above effects of positive grandparenting and the covariates (*F_Model_2a_* (12, 172) = 2.83, *p* = 0.001). For child difficulties examined in Model_2b (*F_Model_2b_* (12, 172) = 3.86, *p* < 0.001), the amount of distant mothering’s extra contribution was insignificant (Δ*R^2^* = 0.05, *p* = 0.084) and no variables of distant mothering displayed a significant association with child difficulties.

#### 3.2.3 Quality of mother-grandparent coparenting relationship as an independent variable

For child prosocial behavior, beyond the contribution of all covariates, positive grandparenting, and distant mothering, the quality of mother-grandparent coparenting relationship accounted for a little more of the variance in this outcome (*F_Model_3a_* (13, 171) = 2.98, *p* < 0.001; Δ*R^2^* = 0.01, *p* = 0.045). Model_3a demonstrates that children tended to be more prosocially when their families reported a higher quality of coparenting relationship between mothers and grandparents (*β* = 0.14, *p* = 0.045). Regarding child difficulties, the coparenting relationship quality did not significantly contribute to explaining the variance of child difficulties over and above the covariates, positive grandparenting, and distant mothering (*F_Model_3b_* (13, 1711) = 3.877, *p* < 0.001; Δ*R^2^* = 0.02, *p* = 0.070).

#### 3.2.4 Covariates

Notably, the frequency of father-child telecommunication was negatively associated with child difficulties in both Model_1b (*β* = −0.15, *p* = 0.038) and Model_2b (*β* = −0.14, *p* = 0.042). This association was marginally significant in Model_3b (*β* = −0.13, *p* = 0.058). The results indicated that children whose migrant fathers more frequently communicated with them through electronic devices showed fewer problem behaviors.

### 3.3 Moderation analyses regarding the role of coparenting relationship quality in the associations between distant mothering/positive grandparenting and child outcomes

The interactions between coparenting relationship quality and each dimension of distant mothering/positive grandparenting were examined separately for child prosocial behavior and child difficulties (Table 4). Almost all interaction terms between coparenting relationship quality and distant mothering/grandparenting for child difficulties were insignificant. There was only one significant interaction effect, i.e., maternal proactive control × coparenting relationship quality, predicting prosocial behavior (Δ*R^2^* = 0.03, *β* = −0.17, *p* = 0.013).

**Table 4:** Moderation models regarding the role of coparenting relationship quality in the associations between distant mothering/positive grandparenting and child outcomes.

Moderation model	Child prosocial behavior as the dependent variable						
	PG × CRQ	RE × CRQ	AG × CRQ	PRC × CRQ	DC × CRQ	PSC × CRQ	PUC × CRQ
Variables	β (SE)	β (SE)	β (SE)	β (SE)	β (SE)	β (SE)	β (SE)
Child age	0.02 (0.07)	0.02 (0.07)	0.01 (0.07)	0.02 (0.07)	0.02 (0.07)	0.02 (0.07)	0.02 (0.07)
Child sex	−0.02 (0.07)	−0.03 (0.07)	−0.03 (0.07)	−0.04 (0.07)	−0.03 (0.07)	−0.04 (0.07)	−0.03 (0.07)
Mother’s years of completed education	0.04 (0.07)	0.03 (0.07)	0.03 (0.07)	0.03 (0.07)	0.03 (0.07)	0.04 (0.07)	0.03 (0.07)
Family income	0.17 (0.07)^*^	0.18 (0.07)^*^	0.17 (0.07)^*^	0.17 (0.07)^*^	0.17 (0.07)^*^	0.17 (0.07)^*^	0.18 (0.07)^*^
Father-child telecommunication frequency	0.12 (0.07)	0.11 (0.07)	0.12 (0.07)	0.11 (0.07)	0.12 (0.07)	0.12 (0.07)	0.11 (0.07)
Positive grandparenting	0.20 (0.07)^**^	0.20 (0.07)^**^	0.20 (0.07)^***^	0.21 (0.07)^**^	0.20 (0.07)^**^	0.18 (0.07)^*^	0.20 (0.07)^**^
Maternal responsivity	0.18 (0.09)^*^	0.17 (0.09)	0.18 (0.09)	0.18 (0.09)^*^	0.18 (0.09)	0.18 (0.09)	0.17 (0.09)
Maternal proactive control	0.08 (0.08)	0.08 (0.08)	0.07 (0.08)	0.09 (0.08)	0.07 (0.08)	0.07 (0.08)	0.08 (0.08)
Maternal directive control	−0.02 (0.08)	−0.02 (0.08)	−0.01 (0.08)	−0.02 (0.08)	−0.01 (0.08)	−0.02 (0.08)	−0.01 (0.08)
Maternal autonomy granting	0.01 (0.09)	0.01 (0.09)	0.01 (0.09)	−0.01 (0.09)	0.01 (0.09)	0.02 (0.09)	0.01 (0.09)
Maternal psychological control	0.05 (0.09)	0.04 (0.08)	0.03 (0.08)	0.06 (0.08)	0.04 (0.08)	0.05 (0.08)	0.04 (0.08)
Maternal harsh punitive control	0.11 (0.09)	0.12 (0.09)	0.11 (0.09)	0.09 (0.09)	0.10 (0.09)	0.12 (0.09)	0.12 (0.09)
Coparenting relationship quality	0.14 (0.07)	0.14 (0.07)^*^	0.14 (0.07)	0.13 (0.07)	0.14 (0.07)	0.13 (0.07)	0.14 (0.07)
Interaction	−0.04 (0.07)	−0.08 (0.07)	−0.05 (0.06)	−0.17 (0.07)^*^	−0.08 (0.07)	0.09 (0.07)	0.06 (0.07)
Δ*R*^2^	0.00	0.01	0.00	0.03^*^	0.01	0.01	0.00

Moderation model	Child difficulties as the dependent variable						
	PG × CRQ	RE × CRQ	AG × CRQ	PRC × CRQ	DC × CRQ	PSC × CRQ	PUC × CRQ
Variables	β (SE)	β (SE)	β (SE)	β (SE)	β (SE)	β (SE)	β (SE)
Child age	0.01 (0.07)	0.01 (0.07)	0.01 (0.07)	0.01 (0.07)	0.01 (0.07)	0.01 (0.07)	0.00 (0.07)
Child sex	−0.12 (0.07)	−0.12 (0.07)	−0.12 (0.07)	−0.12 (0.07)	−0.12 (0.07)	−0.11 (0.07)	−0.12 (0.07)
Mother’s years of completed education	−0.07 (0.07)	−0.07 (0.07)	−0.07 (0.07)	−0.07 (0.07)	−0.07 (0.07)	−0.07 (0.07)	−0.06 (0.07)
Family income	−0.12 (0.07)	−0.12 (0.07)	−0.11 (0.07)	−0.12 (0.07)	−0.12 (0.07)	−0.11 (0.07)	−0.12 (0.07)
Father-child telecommunication frequency	−0.13 (0.07)	−0.13 (0.07)	−0.13 (0.07)	−0.13 (0.07)	−0.13 (0.07)	−0.13 (0.07)	−0.13 (0.07)
Positive grandparenting	−0.25 (0.07)^***^	−0.25 (0.07)^***^	−0.25 (0.07)^***^	−0.25 (0.07)^***^	−0.25 (0.07)^***^	−0.23 (0.07)^**^	−0.24 (0.07)^**^
Maternal responsivity	−0.08 (0.09)	−0.08 (0.09)	−0.08 (0.09)	−0.08 (0.09)	−0.08 (0.09)	−0.08 (0.09)	−0.07 (0.09)
Maternal proactive control	−0.11 (0.08)	−0.12 (0.08)	−0.11 (0.08)	−0.12 (0.08)	−0.12 (0.08)	−0.11 (0.08)	−0.13 (0.08)
Maternal directive control	−0.05 (0.08)	−0.05 (0.08)	−0.05 (0.08)	−0.05 (0.08)	−0.05 (0.08)	−0.05 (0.08)	−0.06 (0.08)
Maternal autonomy granting	−0.02 (0.09)	−0.02 (0.09)	−0.02 (0.09)	−0.01 (0.09)	−0.01 (0.09)	−0.03 (0.09)	−0.01 (0.09)
Maternal psychological control	0.00 (0.08)	−0.01 (0.08)	−0.01 (0.08)	−0.02 (0.08)	−0.02 (0.08)	−0.02 (0.08)	−0.02 (0.08)
Maternal harsh punitive control	0.05 (0.09)	0.04 (0.09)	0.05 (0.09)	0.05 (0.09)	0.05 (0.09)	0.04 (0.09)	0.03 (0.09)
Coparenting relationship quality	−0.13 (0.07)	−0.13 (0.07)	−0.13 (0.07)	−0.12 (0.07)	−0.12 (0.07)	−0.11 (0.07)	−0.12 (0.07)
Interaction	−0.02 (0.07)	0.02 (0.06)	0.02 (0.06)	0.04 (0.07)	0.04 (0.07)	−0.09 (0.07)	−0.11 (0.06)
Δ*R*^2^	0.00	0.00	0.00	0.00	0.00	0.01	0.01

^*^p < 0.05, ^**^p < 0.01, ^***^p < 0.001. PG, positive grandparenting; RE, maternal responsivity; AG, maternal autonomy granting; PRC, maternal proactive control; DC, maternal directive control; PSC, maternal psychological control; PUC, maternal harsh punitive control; CRQ, coparenting relationship quality; SE, standard error. Child sex: 0 = girl, 1 = boy.

**Figure 1:**
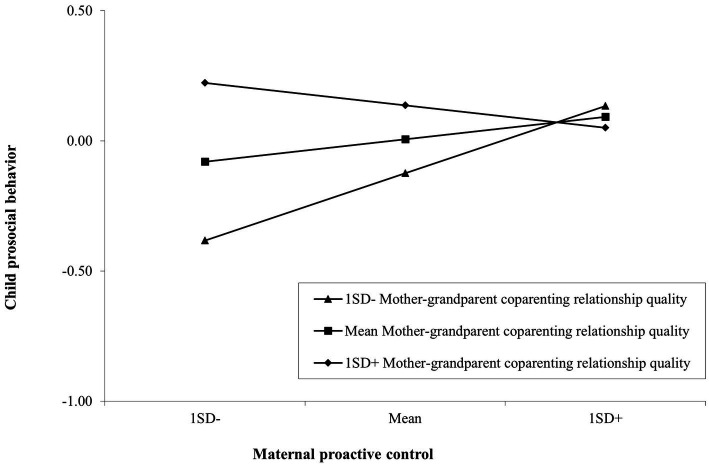
Associations between the mean of maternal proactive control and the mean of child prosocial behavior as a function of low, medium, and high mother-grandparent coparenting relationship.

A *post hoc* test was conducted to interpret the significant interaction effect. Figure 1 depicts the simple regression lines regarding associations between maternal proactive control and child prosocial behavior at low (one *SD* below the mean), medium (mean score), and high (one *SD* above the mean) quality of mother-grandparent coparenting relationship. Only the slope for families reporting low quality of coparenting relationship was significant (*t* = 2.38, *p* = 0.018). The slopes for the mean (*t* = 1.08, *p* = 0.282) and low conditions (*t* = −0.840, *p* = 0.402) were not significant. A low-quality coparenting relationship can be considered a risk factor for child prosocial behavior in families with low levels of proactive control by migrant mothers, given that in this situation, the level of child prosocial behavior was lowest. When mothers used high levels of proactive control, the levels of coparenting quality seemed to make little difference to child prosocial behavior as there was no difference in levels of prosocial behavior between the three groups at low, medium, and high quality of coparenting.

## 4 Discussion

This study examined how distant mothering, positive grandparenting, and mother-grandparent coparenting relationship quality were associated with 3-to-6-year-old children’s prosocial or problem behavior among Chinese rural families characterized by work-related parental migration. Our results based on hierarchical multiple regression analyses showed that: (a) positive grandparenting was positively associated with child prosocial behavior and negatively with child difficulties after considering covariates, distant mothering, and coparenting relationship quality; (b) maternal responsivity through mobile phones showed a unique positive association with child prosocial behavior when controlling for covariates and positive grandparenting; (c) the quality of coparenting relationship was positively related to child prosocial behavior when taking covariates, positive grandparenting, and distant mothering into account; and (d) the association between maternal proactive control and child prosocial behavior depended on the level of coparenting quality, with low levels of maternal proactive control being associated with less child prosocial behavior when the coparenting relationship quality was low, while the coparenting relationship quality did not really matter for child prosocial behavior when mothers used high levels of proactive control.

### 4.1 The role of positive grandparenting in child adjustment

We found congruent evidence to support the importance of positive grandparenting in child development (Weissman et al., 2016; Li et al., 2019; Yang and Liu, 2020), especially for LBC in China (Hou et al., 2019), with results demonstrating that children showed more prosocial behavior and fewer adaptive difficulties when their grandparents used more positive parenting practices (i.e., a combination of warmth, democratic participation, and reasoning practices). The result aligns with our *Hypothesis 1*. The role of positive grandparenting for child adjustment in parent–child-separated families remained significant even when other variables regarding distant mothering and coparenting were taken into account. Grandparents are usually the first primary caregivers in families without parental physical presence. This finding aligns with the idea of the importance of the proximal environment and frequent interaction processes for child functioning and development, outlined in Bronfenbrenner’s bio-psycho-social model (Bronfenbrenner and Morris, 1998). In the Chinese cultural context, norms of filial piety and intergenerational obligation (Yeh and Bedford, 2003) also legitimize grandparents’ central caregiving role and authority in the family, which may further strengthen the impact of their positive grandparenting practices on grandchildren’s adjustment. In terms of grandparents’ protective role, their warmth and support can contribute to the psychological resilience of grandchildren and buffer against the influence of adversities in grandchildren’s life, consequently following a decreased risk of socioemotional disorders (Dunifon, 2013; Li et al., 2021). Additionally, from the perspective of the facilitating role of grandparents (Paulus and Moore, 2012), grandchildren might learn to use prosocial skills in family and preschool social occasions from grandparents’ democratic interactions with them and appropriate control by reasoning.

### 4.2 The associations between distant mothering practices and child adjustment

For bivariate associations between distant mothering dimensions and child adjustment without including covariates, as expected, child prosocial behavior was positively correlated with maternal responsivity and maternal autonomy granting. Child difficulties were negatively correlated with maternal responsivity, maternal autonomy granting, and maternal proactive and directive control while positively correlated with maternal psychological control and harsh punitive control. When also considering positive grandparenting and demographic variables, no evidence of hypothesized links between dimensions of distant mothering and child adjustment was detected, except for the finding that maternal responsivity was significantly positively related to child prosocial behavior. The findings provide partial support for our *Hypothesis 2*.

Although several distant mothering dimensions showed associations in the expected directions at the bivariate level, the zero-order correlations with child outcomes were modest (|*r*| = 0.06–0.24), which is comparable to effect sizes typically reported for general parenting (|*r*| = 0.01–0.24; Pinquart, 2017a, 2017b; Wong et al., 2021). Given these small-to-modest effect sizes, the relatively small triadic sample and the number of predictors included in the regression models, the present study may have been underpowered to detect unique effects of autonomy granting and proactive or directive control once positive grandparenting and other covariates were taken into account. Positive grandparenting also accounted for a substantial proportion of variance in prosocial behavior and overlaps conceptually with more supportive and structured distant mothering practices, which likely reduced the unique variance that could be attributed to the other positive distant mothering dimensions, even though multicollinearity diagnostics were acceptable. In this context, distant responsivity may represent the most distinctive aspect of maternal communication via mobile phones, conveying warmth and contingent emotional support, which could explain why it emerged as the only significant predictor in the multivariate models. It is also possible that the other distant mothering dimensions play a role in child adjustment primarily through indirect or child-driven pathways. For example, they may shape other family subsystems (such as coparenting or grandparenting) or be adjusted in response to children’s existing behavior. Thus, they may not emerge as strong independent predictors in our cross-sectional models.

The results suggested that children whose mothers used more responsivity in their audio or video calls also tended to be more prosocial in homes and preschools, in line with previous research on general maternal parenting (Garner, 2006; Wang et al., 2006; Pastorelli et al., 2016). Maternal responsivity can be related to child prosocial behavior in various ways. Attachment researchers argue that emotional warmth provided by mothers makes children feel secure and trusted, subsequently developing children’s sense of belonging and initiative of connecting with others (Ferreira et al., 2016). Also, mothers’ support and warmth practices can inform children of the model of sympathetic engagement, caring, and helping others (Paulus and Moore, 2012). Moreover, children may be more willing to internalize their mothers’ values regarding respect and connectedness to others when they feel the warmth from their mothers (Grusec and Goodnow, 1994). Consistent with these interpretations, our earlier qualitative study on mobile-phone-based parenting (Liang et al., 2023) showed that migrant mothers deliberately tried to use praise, comfort, and contingent responses during calls to convey warmth and support to their LBC.

However, the role of maternal distant responsivity in explaining child prosocial behavior was rather limited. This suggests that compared to other factors like genetics, temperaments, peers, preschools, and grandparenting, distant mothering may not be the most important aspect in the complex process of LBC’s early childhood socioemotional behaviors. A bidirectional influence between mothering and child behaviors (Pastorelli et al., 2016) might also complicate the examination of the individual contribution of distant mothering, as bidirectional relationships increase the difficulty in isolating the individual contribution of the independent variable which may change with the dependent variable in feedback loops. From a child-driven perspective, children’s existing prosocial tendencies and behavior problems may themselves shape how mothers behave during calls: more prosocial and easy-to-manage children may evoke warmer, more responsive interactions, whereas children who show more difficulties may elicit more controlling or even harsher responses. This interpretation is supported by our earlier qualitative study on mobile-phone-based parenting (Liang et al., 2023), in which migrant parents reported adjusting the frequency, length, and content of phone calls according to children’s mood, cooperation, and well-being, and described how some children actively requested or initiated contact with their parents. In this case, the parenting practices we observe partly reflect mothers’ responses to children’s current behavior, which can weaken or hide the direct effect of distant mothering on child adjustment in cross-sectional analyses.

On the one hand, concurrent associations between distant responsivity and child prosocial behavior may accumulate over time to demonstrate a more noticeable impact if mother–child separation is prolonged. On the other hand, as a subsystem in a family, distant mothering can interact with other parts of family dynamics (e.g., grandparenting, Acedera and Yeoh, 2021) and further indirectly impact child development and well-being.

### 4.3 The associations between intergenerational coparenting relationship quality and child adjustment

The quality of mother-grandparent coparenting relationship was positively correlated with child prosocial behavior and negatively correlated with child difficulties. When taking demographic characteristics, positive grandparenting and distant mothering into account, the quality of mother-grandparent coparenting relationship still contributed to the variance of child prosocial behavior. In line with part of *Hypothesis 3*, the present findings showed that children from families with a higher quality of mother-grandparent coparenting relationship showed more prosocial behavior at home and in preschool. This result is consistent with previous research that has demonstrated positive associations between father–mother/intergenerational coparenting quality and child emotional and social competence (Teubert and Pinquart, 2010; Li and Liu, 2019; Xu et al., 2023). Family systems theory underlines the jointly-created family climate and the interdependence of family members’ feelings, values, and behaviors (Minuchin, 1985; Cox and Paley, 1997). Accordingly, children in our study might be more likely to replicate the communicative patterns between mothers and grandparents through role modeling processes to develop their prosocial tendencies if these coparenting figures frequently showed harmonious reciprocities, effective telecommunications, shared values, cooperation, and mutual support during distant interactions by electronic devices. Moreover, the prevention of situations where mothers and grandparents compete for the closeness of their (grand)child or where grandparents blame migrant mothers for the shifted childrearing duties, may promote child prosocial behavior by increasing the child’s sense of stability and security (Lau, 2017). From a cultural perspective, filial piety norms may encourage adult children to respect and defer to grandparents’ views on childrearing, which can facilitate cooperative coparenting when values are aligned but also make it difficult for migrant mothers to explicitly challenge grandparental practices when disagreements arise. Such dynamics may partly shape how mother-grandparent coparenting quality is experienced in LBC’s families.

Inconsistent with part of *Hypothesis 3*, the current study did not find any significant association between coparenting relationship quality and child difficulties. This may be explained by the indirect paths from mother-grandparent coparenting quality to child problem behaviors through other parenting characteristics like maternal sensitivity (Liang et al., 2021). In other words, despite the statistically insignificant link, the quality of mother-grandparent coparenting relationship may remain critical for other facets of child adjustment like child difficulties. The importance of the coparenting relationship quality in child adaptation will be gradually confirmed as future studies further include more factors of the parenting process and reveal underlying indirect mechanisms among the subsystems within a family.

### 4.4 The role of the interactions between coparenting relationship quality and distant mothering dimensions in child adjustment

Although we found that the quality of mother-grandparent coparenting relationship moderated the association between distant proactive control by mothers and child prosocial behavior, the interaction between coparenting relationship quality and proactive control was not a synergistic or enhancing pattern where the joint effects of positive mothering and good quality of coparenting relationship were greater than the total of their individual effects as previous research identified (Scrimgeour et al., 2013; Yan et al., 2021). The findings partially diverge from *Hypothesis 4*. Instead, our results showed the interaction had an either-or effect on the association between maternal proactive control and child prosocial behavior. This either-or effect means that the significance of the relationship between the independent variable and the dependent variable changes according to the level of the moderator (Cohen et al., 2013). In our study, children from families with both low levels of mother-grandparent coparenting relationship quality and maternal proactive control showed the lowest level of prosocial behavior, whereas there was no significant difference in levels of prosocial behavior of children at any levels of coparenting quality as long as mothers reported high levels of maternal proactive control. In other words, the risk of low quality of mother-grandparent coparenting relationship for child prosocial behavior was salient at low levels of proactive control by migrant mothers while the risk seemed to be negligible when mothers used high levels of proactive control. However, this interaction effect should be interpreted with caution, as it did not remain statistically significant after adjusting for multiple comparisons using Bonferroni and Holm-Bonferroni corrections.

Considering the multiple family-related determinants of child prosocial tendencies (Eisenberg and Mussen, 1989), we can speculate that the extent to which coparenting relationship quality and distant mothering were related to child prosocial behavior might vary in different families. Young children are selective learners in the social learning process and they can decide what and from whom to learn based on social cues like the competence of the individuals and reinforcements/rewards (Poulin-Dubois and Brosseau-Liard, 2016; Heyes, 2017). In families with medium- or high-quality coparenting relationships, children might be likely to benefit more from the modeling of cooperative and positive communications between migrant mothers and grandparents than from mother–child telecommunications. This interpretation is consistent with our earlier qualitative study (Liang et al., 2023), in which some migrant parents described how harmonious or conflictual relationships with at-home caregivers shaped the way they used mobile-phone-based guidance and discipline in everyday situations. When mothers and grandparents cannot achieve a relatively cooperative and supportive relationship and have more conflicts in childrearing, LBC might rely more on mothers’ appropriate disciplinary techniques to learn altruistic actions if their mothers show high levels of proactive control. It is possible that in the case of low quality of intergenerational coparenting relationship and high levels of maternal proactive control, the children gained more material or emotional rewards from their mothers, or they believed that their mothers were more reliable and competent figures to imitate. Mothers’ explanations, reasoning, verbal persuasion, and preaching are all proactive control behaviors, by which children can model being considerate and rational for social interactions and get information about unwanted social behaviors and the awareness of being responsible for their own behaviors (Eisenberg and Mussen, 1989; Pastorelli et al., 2016).

The study was conducted in Chuxiong Yi Autonomous Prefecture, where a substantial proportion of the population belongs to Yi and other ethnic minority groups. At the same time, Yi and Han families in this region typically live in mixed and relatively integrated communities. Although we did not collect individual-level data on participants’ ethnic identity, it is likely that our sample included both Yi and Han families. Existing work suggests that ethnic minority communities in these areas may place a strong emphasis on extended family networks, grandparental involvement in childcare, and intergenerational obligations (Chen and Zhan, 2012), which could contribute to the prominent role of grandparents and the patterns of intergenerational coparenting observed in this study. However, without direct ethnicity measures and without in-depth anthropological data on the specific communities we studied, it is difficult to disentangle the influence of Yi cultural traditions from broader rural childrearing norms in southwest China. Our discussion on cultural influences should therefore be seen as tentative.

### 4.5 The underexplored but noteworthy role of distant fathering in child adjustment

In addition to the main aims of the current study, it is worth noting that more frequent father-child telecommunication was related to fewer child difficulties after taking into account other demographic features, positive grandparenting, and distant mothering. This is consistent with the results of previous research that found the positive influence of fathers’ involvement on internalizing and externalizing symptoms of Chinese LBC (Wu et al., 2021). Although distant fathering was not a primary focus in this substudy and was included only as a covariate, this association suggests that regular contact with migrant fathers may also act as a protective resource for LBC’s socio-emotional adjustment and warrants more systematic examination in future research.

### 4.6 Strengths and limitations

This study was innovative, because we combined the perspectives of intergenerational coparenting and coparenting in the context of family separation and media use, which provided a new framework for LBC’s family education and intervention. Furthermore, the multi-informant reports in our study provided a more comprehensive picture of children’s behaviors across different settings (home and preschool) and the ratings of coparenting relationships by both mothers and grandparents increased validity and mitigated bias.

Several limitations in the current study could be addressed in the future. First, the modest sample size limited our ability to detect interaction effects with adequate statistical power. In additional analyses, the interaction term between maternal proactive control and coparenting relationship quality did not remain significant after adjusting *p*-values for multiple comparisons (Bonferroni and Holm-Bonferroni corrections). Therefore, the moderating effect reported in this study should be regarded as tentative and interpreted with caution. Second, this study focused only on positive grandparenting and the overall coparenting relationship, as measured by the short version of the CRS. Consequently, we did not differentiate between specific aspects of grandparenting (e.g., gender-biased caregiving, harsh punishment) or coparenting (e.g., coparenting support, coparenting undermining). In addition, the quality of a more general relationship between grandparents and migrant parents is also valuable to include in future research, because intergenerational solidarity, which is affected by new media and communication technologies, is important for family functioning (Taipale, 2019). Other aspects of intergenerational relationships (e.g., Grandparent’s satisfaction with the support provided by their adult children in using communication devices.) may be associated with parenting behaviors and child well-being. Third, because the number of participants in the study was limited, we could not differentiate the effects between mother-maternal grandmother, mother-paternal grandmother, mother-maternal grandfather, or mother-paternal grandfather coparenting patterns. It was thus not possible to compare group differences in the coparenting relationship-child adjustment associations among these dyads, as He et al. (2023) did. Forth, although we noticed the importance of father-child telecommunication in terms of frequency, this study did not include the quality of distant fathering, which may play a unique role in LBC’s well-being and development. Fifth, this study adopted a cross-sectional design to explore relationships between the variables of our concern, and thus cannot reveal causality and accumulative long-term effects of grandparenting, distant mothering, and coparenting relationship quality on child adjustment. Additionally, such cross-sectional design, conducted during the COVID-19 pandemic, needs replication to see whether the findings apply also in post-pandemic conditions. Sixth, child adjustment in this study was operationalized as child prosocial behavior and difficulties, and we could not test whether distant parenting practices are associated with other possible child outcomes, such as child emotional regulation, self-esteem, and well-being. Seventh, the small sample size, suboptimal sampling methods, the specific study context, and the absence of follow-up qualitative work with both parents and grandparents may limit the generalizability and depth of our findings. Eighth, we did not collect information on some potentially relevant family characteristics, such as sibling status or ethnic background. Future studies with larger samples and more extensive demographic assessments should include sibling characteristics and ethnicity to provide a more comprehensive and contextually sensitive picture of LBC’s family environments.

## 5 Conclusion

In conclusion, the study highlights the significant role of positive grandparenting in helping young left-behind children in rural China better adapt both psychologically and socially. Migrant mothers and in-hometown grandparents can build a coparenting relationship from a distance. It also emphasizes the importance of a high-quality mother-grandparent coparenting relationship for supporting child prosocial behavior, while noting that maternal responsivity played a less noticeable but meaningful role in this child outcome. Additionally, the study underscores the importance of dynamics between family subsystems for child adaptation. The quality of the mother-grandparent coparenting relationship moderated the association between mothers’ distant proactive control and child prosocial behavior.

### 5.1 Implications for practices

Given the identified unique functions of grandparenting and distant parenting for psychosocial and behavioral adjustment of LBC, separate intervention programs aiming at grandparent caregivers or migrant parents turned out effective (Chan et al., 2019; To et al., 2019) and can be recommended to stakeholders. If possible, an individualized video self-modeling approach (Regan and Howe, 2017) can be further used in families with young LBC at a higher risk of adaptive problems, to improve grandparent–child or parent-child interactions. Practitioners can use the items in the authoritative style subscale of the SRDQ and the responsivity subscale of the MPPPQ to discuss parenting practices with those caregivers.

In addition, a comprehensive family intervention program involving parents and grandparents at the same time may be a better option given the interrelatedness of (behaviors of) members within different family subsystems. Considering the risk of less child prosocial behavior in families with low-quality mother-grandparent coparenting relationships and low levels of maternal proactive control, practitioners can, if necessary, pay more attention to this group and additionally provide them with information about proactive control in distant parenting. Besides the focus on parenting practices, the integrative framework of intergenerational coparenting proposed by Bai et al. (2023) and the framework by Feinberg (2003) can inform these interventions to consider including diverse aspects that aim to establish a shared understanding among different generations regarding power dynamics, authority, and division of labor. For interventions conducted in familism-oriented societies, some researchers emphasize considering the importance of filial piety in project development (Bai et al., 2023).

## Data Availability

The raw data supporting the conclusions of this article will be made available by the authors, without undue reservation.
